# Age and Body Condition Influence the Post-Prandial Interleukin-1β Response to a High-Starch Meal in Horses

**DOI:** 10.3390/ani11123362

**Published:** 2021-11-24

**Authors:** Jessica Suagee-Bedore, Nichola Shost, Christian Miller, Luis Grado, Jeremy Bechelli

**Affiliations:** 1 School of Agricultural Sciences, College of Science and Engineering Technology, Sam Houston State University, Huntsville, TX 77340, USA; NLS034@shsu.edu; 2 Department of Biological Sciences, College of Science and Engineering Technology, Sam Houston State University, Huntsville, TX 77340, USA; cjm110@shsu.edu (C.M.); lag042@shsu.edu (L.G.); jrb138@shsu.edu (J.B.)

**Keywords:** equine, inflammasome, interleukin-1β, non-structural carbohydrates

## Abstract

**Simple Summary:**

In horses, consumption of meals rich in nonstructural carbohydrate content transiently increase plasma concentrations of the pro-inflammatory cytokine interleukin-1β. The current experiment provides evidence that age and body condition score influence these results. For instance, younger and leaner horses only experienced this response after regular and prolonged intake of high-starch meals, whereas older and heavier conditioned individuals experience elevated post-prandial interleukin-1β concentrations on day 1 of feeding and thereafter.

**Abstract:**

Older horses and those prone to obesity may be at a higher risk for inflammation than younger and leaner counterparts. Previous research indicated a postprandial elevation in plasma concentrations of interleukin-1β (IL-1β), a pro-inflammatory cytokine, after consuming 1.2 g of non-structural carbohydrates/kilogram of body weight. However, these studies utilized horses of mixed age and body condition. The current study evaluated post-prandial IL-1β concentrations in horses specifically comparing lean to over-conditioned and middle aged to older. Our results suggest that at least two weeks of daily consumption of a high non-structural carbohydrate diet is required to induce a post-prandial increase in IL-1β concentrations in younger and leaner horses. In opposition to this, older and over-conditioned horses experience plasma increased on the first day of feeding and thereafter. Feeding management practices of older and over-conditioned individuals should emphasize lower non-structural carbohydrate intakes and further research should elucidate mechanisms of IL-1β activation.

## 1. Introduction

The inflammasome is a multi-protein complex consisting of nucleotide-binding oligomerization domain and leucine-rich repeat-containing receptors (NLRs) and the protease caspase-1 that forms in response to intracellular pathogens and danger signals [1]. In response to various stimulants and/or danger signals including adenosine triphosphate (ATP), pathogen-associated molecular patterns (PAMPS), damage-associated molecular patterns (DAMPS), or various environmental irritants, the activation of NLRs oligomerize the adaptor protein apoptosis-associated speck-like protein containing caspase activation and recruitment domain (CARD) (ASC), which triggers the proteolytic activity of caspase-1. Activated caspase-1 then mediates cleavage of pro-interleukin (IL)-1β and pro-IL-18 into biologically active IL-1β and IL-18 [2]. Inflammasome activation is considered to be a two-signal pathway, whereby expression of nucleotide oligomerization domain (NOD)-, leucine rich repeat (LRR)- and Pyrin domain-containing protein 3 (NLRP3) and pro-IL1β are increased following activation of nuclear factor κ B (NFκB) and then a second signal is required to activate the scaffolding of NLRP3.

**Figure 1:**
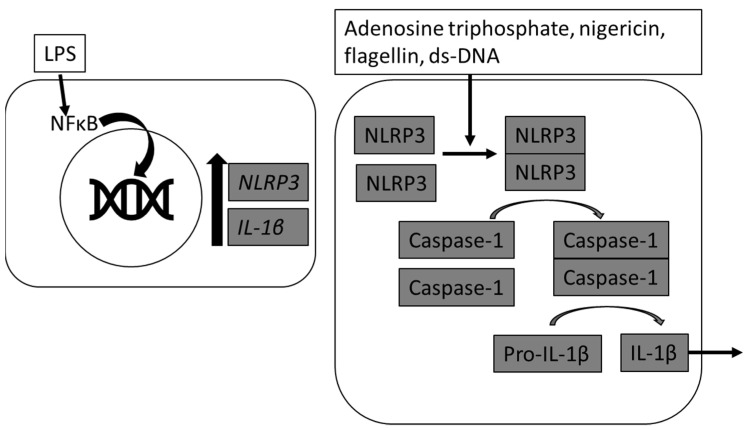
Mechanism of inflammasome activation of interleukin (IL)-1β. In the first step, lipopolysaccharide (LPS) primes cells by increasing *NLRP3* and *IL-*1β mRNA abundance. In the second step, adenosine triphosphate, nigericin, flagellin, and ds-DNA can induce the inflammasome to assemble, activating caspase-1, and maturating IL-1β. Mature IL-1β can be secreted from the cell.

The first signal step of NFκB activation can be achieved through a variety of microbial products and ligands, including lipopolysaccharide (LPS), which increase mRNA abundance of NLRP3, as shown in rodent and human models [3] (Figure 1). The inflammasome is then activated to assemble by a wide array of infectious and non-infectious pathways, including the Gram-positive bacterial toxin nigericin, extracellular ATP, and reactive oxygen species [4,5]. In equine peripheral blood monocytes (PBMC) NLRP3 inflammasomes are primed by LPS and triggered to produce mature IL-1β in response to extracellular ATP, nigericin, flagellin, and ds-DNA [6].

Horses have a mild and transient increase in plasma concentrations of interleukin-1β (IL-1β) in response to consuming a meal that is higher in starch and sugar (non-structural carbohydrates; NSC). Our previous work has demonstrated this finding in Thoroughbred mares consuming 1.14 g NSC/kg bodyweight and in mixed breed geldings consuming 1.2 g NSC/kg bodyweight [7,8]. Furthermore, we have also previously shown that blood concentrations of LPS are increased at 2 h post meal consumption in horses [9], suggesting inflammasome priming could be occurring in the post-prandial state.

Previous research in horses has indicated interacting factors of age and body condition score (BCS) on inflammatory responses. For instance, higher concentrations of inflammatory proteins, even in an unstimulated state, have been documented in horses older than 20 years [10]. Regarding obesity, Reynolds et al. [11] demonstrated increased adipose tissue gene expression of *IL-1β* in obese horses. For these reasons we planned a preliminary investigation into the effects of age and body condition score on the post-prandial IL-1β response to NSC consumption. While original studies used compounded feeds with higher levels of NSC, these types of feeds are now difficult to source through commercial vendors as they are unpopular in the American marketplace. Therefore, we chose to use steam-rolled barley, a processed grain with high endogenous NSC concentrations that have been shown to alter microbial populations in the equine intestine and increase lactic acid production [12]. Based on studies in ruminants, these factors are implicated in the production and appearance of LPS in the blood [13]. Therefore, we hypothesized that horses older than 20 and those with higher BCS would have greater IL-1β responses to NSC consumption than their younger or lean counterparts.

## 2. Materials and Methods

### 2.1. Trial 1 Animals and Treatments

The use of animals for this research was approved by The Ohio State University’s Institutional Animal Care and Use Committee. Ten horses were used for this study, consisting of six Quarter Horses, two warmbloods, and two Standardbreds. They ranged in body weight from 468 to 651 kg, 10 to 17 years of age, and included 4 geldings and 6 mares. Horses were assigned to treatment based on BCS, with lean horses having a BCS between 4 and 5 (*n* = 5; 468–532 kg) and overweight horses having a BCS between 6.5 and 8 (*n* = 5; 500–651 kg). The BCS scale was that of Henneke et al. [14], which ranks horses from 1 to 9, with 1 indicating emaciation and 9 indicating obesity. Sample size was determined based on a power-analysis of previous data demonstrating an increase in post-prandial IL-1β concentrations in non-obese horses.

Horses were group-housed in an outdoor paddock with access to ad libitum mixed grass hay (Table 1), salt, and water. Horses received treatment diets for 14 d, with steam-rolled barley (SRB; Table 1) being fed between 07:00 and 08:00 a.m. and a ration balancer (Essential K, Kalmbach Feeds, Sandusky, OH, USA; 0.45 kg per day, Table 1) being fed between 15:00 and 16:00 p.m. Horses were fed in individual pans spread at a distance to prevent sharing of feed and were monitored throughout feeding sessions. Steam-rolled barley was fed at a rate to provide 1.2 g of NSC per kg BW.

On days 0 and 13, horses were relocated from paddock to stalls at 16:00 p.m. and provided ad libitum water and 1 kg of their hay. On the following mornings, horses were offered their SRB meal and had blood collected at –30 min (07:30 a.m.), and 1, 4, and 8 h post-feeding. Horses received 2 kg of their grass hay after the 1 h sample collection. Blood was centrifuged at 1500× *g* for 10 min to separate plasma. Plasma was separated and stored at −20 °C until analysis for concentrations of IL-1β.

### 2.2. Trial 2 Animals and Treatments

Sam Houston State University’s Institutional Animal Care and Use Committee approved the use of animals for this research. Six horses were used for this study, consisting of two Thoroughbreds, two Tennessee Walking Horses, and two Quarter Horses. Of these horses, there were three geldings and three mares, all with body condition scores between 5 and 6. Horses were grouped by age, where aged horses ranged from 20–23 years (OLD; 514–582 kg), and middle-aged horses ranged from 12–14 years (MID; 455–500kg). Sample size was determined based on a power-analysis of previous data demonstrating an increase in post-prandial IL-1β concentrations in non-obese horses.

Horses were fed in individual stalls, with access to coastal bermudagrass hay (*Cynodon dactylon*), fed at 2% of bodyweight (Table 1), and ad libitum salt and water. Horses received treatment diets with steam-rolled barley being fed between 07:00 and 08:00 a.m. and a commercial concentrate (SafeChoice Original, Cargill Animal Nutrition, Wayzata, MN, USA, Table 1). Steam-rolled barley was fed at a rate to provide 1.2 g of NSC per kg BW. The commercial concentrate was fed at a rate of 0.5% bodyweight and split between two feedings; one feeding followed barley consumption, and the other occurred between 15:00 and 16:00 p.m. Horses received the barley diet for 36 days, a longer time frame than Trial 1, to enable a greater chance of capturing gene expression changes.

**Figure 2:**
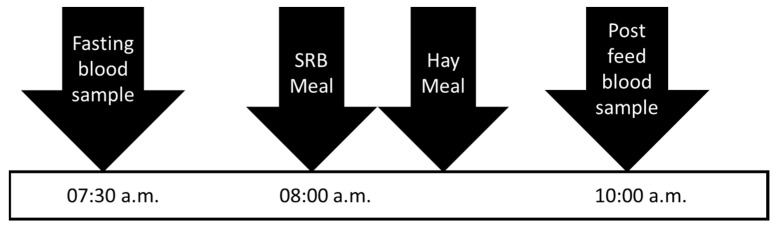
Timeline of sample collection during trial 2. Horses were fasted for at least 11 h prior to collection of fasting blood at 07:30 a.m. A meal of steam rolled barley (SRB) that provided 1.2 g nonstructural carbohydrates per kilogram of bodyweight was offered at 08:00 a.m. with hay offered once grain was consumed. A second blood sample was collected at 10:00 a.m.

On days 0 and 35, horses were observed to have finished their evening hay by 20:00 p.m. and were not provided additional hay until sample collection the following morning. On days 1 and 36, horses were offered their SRB meal and had blood collected at –30 min (07:30 a.m.), and 2 h post-feeding (Figure 2). Horses received their hay allotment after they finished consuming their SRB. Blood was centrifuged at 1500× *g* for 10 min to separate plasma. Plasma was separated and stored at −20 °C until analysis for concentrations of IL-1β. White blood cells from whole blood were subject to RNA extraction using the PureLink^TM^ Total RNA Blood Kit (K156001; ThermoFisher Scientific, Waltham, MA, USA) according to the manufacturer’s instructions.

### 2.3. Sample Analysis

Plasma was analyzed for concentrations of IL-1β using the Equine IL-1β Do-It-Yourself ELISA (DIY0699E-003; KingFisher Biotech, St. Paul, MN, USA) according to previously published methods [8]. Briefly, Nunc Maxisorb 96-well plates (VWR, Radnor, PA, USA) were coated with capture antibody at a concentration of 3 µg/mL in phosphate-buffered saline (Product # 28374; BuPH^TM^ Modified Dulbecco’s PBS Packs, ThermoFisher Scientific, Waltham, MA, USA) and allowed to incubate overnight at 4 °C. Plasma was diluted 1:2 with reagent diluent, which consisted of 4% bovine serum albumin (Product # 126593; Bovine Serum Albumin, Fraction V, EMD Millipore Sigma, Burlington, MA, USA) in PBS, and incubated for one hour at room temperature. The detection antibody was diluted to a concentration of 3 µg/mL in reagent diluent. Optical density values were obtained using a plate reader (SpectraMax, Molecular Devices, San Jose, CA, USA) and converted to concentrations using the plate reader’s software (SoftMax Pro, VWR, Radnor, PA, USA).

To determine host gene expression, horse blood was collected in lithium heparin tubes. Total RNA was extracted via PureLink™ Total RNA Blood Purification Kit (K156001; Invitrogen, Carlsbad, CA, USA), and cDNA was synthesized utilizing Protoscript First Strand cDNA Synthesis Kit (E6300S; New England Biolabs, Ipswich, MA, USA) following the standard protocol guide. qRT-PCR assays were performed using Luna Universal Probe qPCR Master Mix in 20 μL volumes according to the manufacturer’s instructions (New England Biolabs, Ipswich, MA, USA). Cycling conditions were set for an initial denaturation of 95 °C for 60 s for one cycle, and denaturation of 95 °C for 15 s and extension of 60 °C for 30 s for 40 cycles. To check specificity of amplification, melt curve analysis was performed on each amplification. Transcript abundance was calculated utilizing the 2^−ΔΔCT^ method and normalized to glyceraldehyde-4-phosphate dehydrogenase (GAPDH). Primers used in qRT-PCR analysis are listed in Table 2.

### 2.4. Statistical Analysis

All statistical analyses were conducted using the SAS^®^ OnDemand for Academics webserver (2021 SAS Institute, Cary, NC, USA). For all repeated measures analyses, the covariance matrix yielding the lowest AICC was used. For trial 1, plasma concentrations of IL-1β were analyzed by repeated measures analysis of variance (ANOVA) for the effects and interactions of day (1 vs. 14) and hour (0, 1, 4, and 8) within treatment (overweight vs. lean). Hour within day was the repeated term. Hour 0 concentrations were used as a covariate after confirming significance, and all values were log_10_ transformed to improve normality and homogeneity of variance. A Dunnett test was used to determine significance of simple effects, which is a test that compares each time point to time 0 only. For trial 2, plasma concentrations of IL-1β and fold change mRNA abundance were analyzed by repeated measures ANOVA for the effects and 2 way-interactions of day (1 vs. 36), hour (0 vs. 2), and age (12–14 years vs. 20–22 years). When 2 × 2 interactions were considered significant, specific contrasts were used to compare simple effects.

## 3. Results

### 3.1. Trial 1

**Figure 3:**
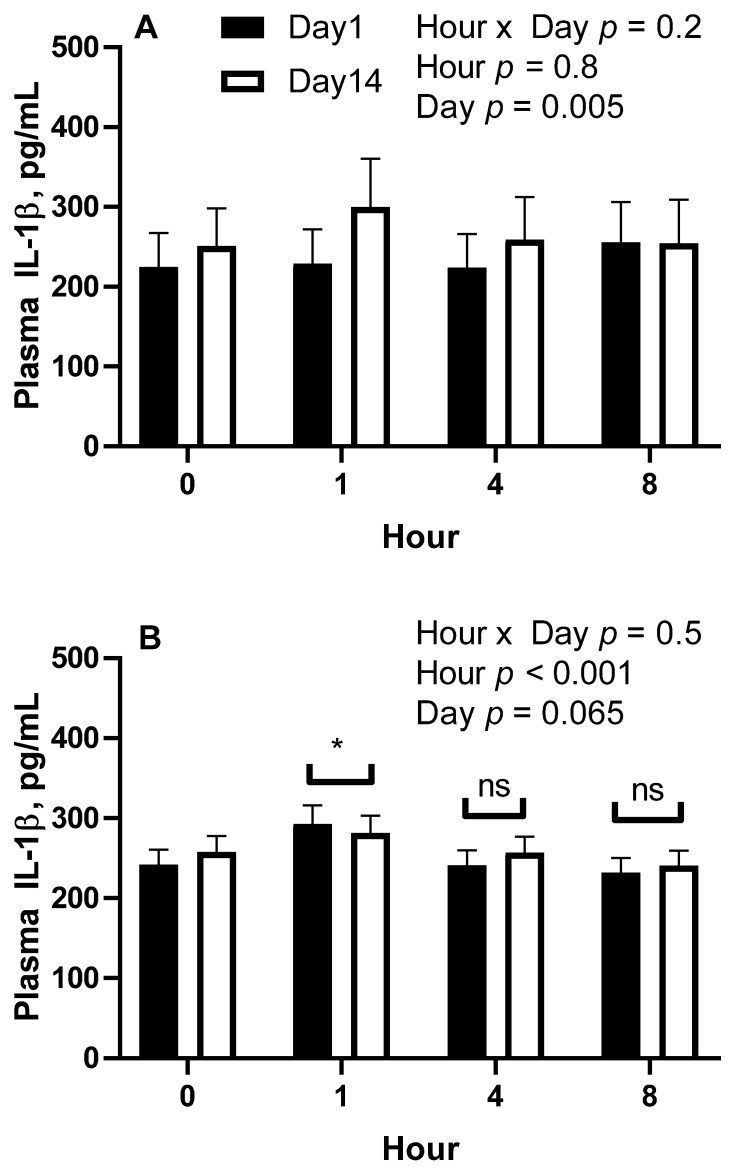
Geometric mean (±95% confidence interval) plasma interleukin-1β concentrations in lean (**A**); body condition score 4-5, *n* = 5) and overweight (**B**); body condition score 6-7, *n* = 5) on day 1 and 14 of receiving a meal of steam flaked barley providing 1.2 g of nonstructural carbohydrates per kilogram of bodyweight per meal, where blood samples were collected after an overnight fast (hour 0) and then 1, 4, and 8 h post-feeding. * Geometric means are different from hour 0, *p* < 0.05.

For trial 1, residuals of IL-1β concentrations were not normally distributed; therefore, data were log-transformed before analysis. Means were back-transformed after analysis and are presented as geometric means and 95% confidence intervals. Data from body condition score groups were analyzed for the effect of hour post-feeding (hour) and day (1 vs. 14) and the interaction of hour and day. In lean horses there was an effect of day, whereby geometric mean plasma IL-1β concentrations were higher on day 14 (265 [251, 281] pg/mL) than on day 1 (233 [222, 245] pg/mL; *p* = 0.005; Figure 3). There were no effects of hour or hour-by-day interaction (*p* > 0.2). For overweight horses, there was an effect of hour (*p* < 0.001; Figure 3), whereby geometric mean concentrations were higher at 1 h post-feeding (287 [272, 303] pg/mL; *p* = 0.010) than prior to feeding (250 [237, 263] pg/mL). Plasma concentrations at hours 4 (249 [236, 262] pg/mL and 8 (236 [224, 249] pg/mL) post-feeding were not different (*p* > 0.1) than pre-feeding concentrations. There was a tendency for plasma IL-1β concentrations to be higher (*p* = 0.065) on day 14 (258 [253, 264] pg/mL) than day 1 (251 [245, 257] pg/mL). There was no effect of the hour-by-day interaction for overweight horses (*p* > 0.5).

### 3.2. Trial 2

**Figure 4:**
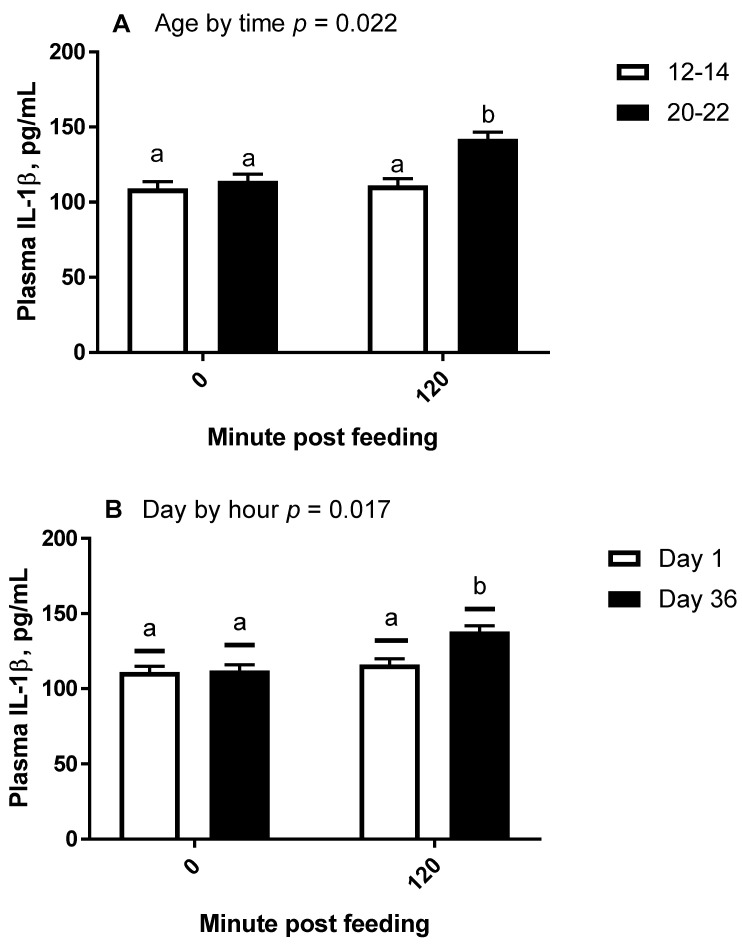
Geometric mean plasma (±95% confidence interval) interleukin-1β concentrations in horses receiving a meal of steam flaked barley providing 1.2 g of nonstructural carbohydrates per kilogram of bodyweight per meal, where blood samples were collected after an overnight fast (hour 0) and at 120 min post-feeding. Differences in concentrations are presented by age (**A**) whereby horses were either middle aged (12–14 years, *n* = 3) or older (20–22 years, *n* = 3), and by day of study (**B**) whereby IL-1β concentrations are reported on day 1 and day 36 of barley consumption. ^ab^ Unlike superscripts indicates geometric means differ *p* < 0.05.

For trial 2, residuals of IL-1β concentrations were found to be normally distributed and homogenous, therefore are presented as means ± SEM. There was an interaction of day by time (*p* = 0.017; Figure 4) whereby plasma IL-1β concentrations were higher on day 36 post-feeding (136 ± 4 pg/mL) than day 36 pre-feeding (112 ± 4 pg/mL; *p* = 0.002). Day 36 post-feeding concentrations were also higher than either day 1 pre-feeding (111 ± 4 pg/mL; *p* = 0.002) or post-feeding (116 ± 4 pg/mL; *p* = 0.008). Day 1 pre- and post-feeding concentrations were also not different from each other (*p* > 0.8). There was also an age by time interaction (*p* = 0.022; Figure 4) whereby plasma IL-1β concentrations were higher in old horses post-feeding (142 ± 5 pg/mL) than pre-feeding (114 ± 5 pg/mL; *p* = 0.012) and were also higher than middle aged horses both pre (109 ± 5 pg/mL; *p* = 0.008) and post-feeding (111 ± 5 pg/mL; *p* = 0.002). In middle-aged horses, pre- and post-feeding IL-1β concentrations were not different (*p* > 0.9). Lastly, there was no effect of the age-by-day interaction (*p* > 0.6).

**Figure 5:**
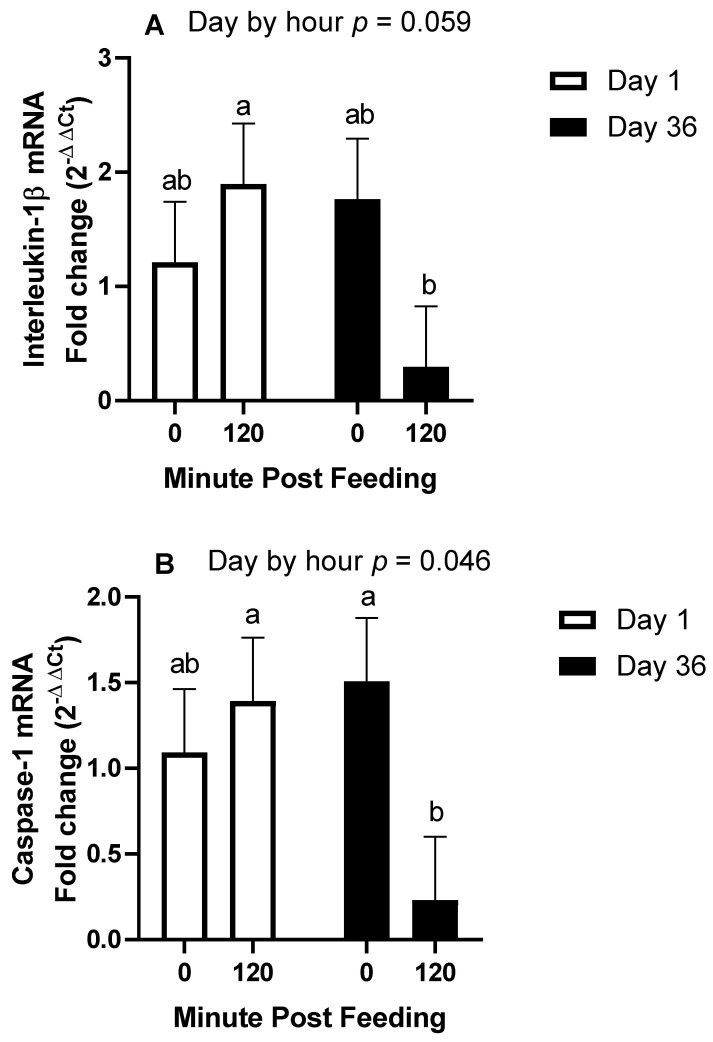

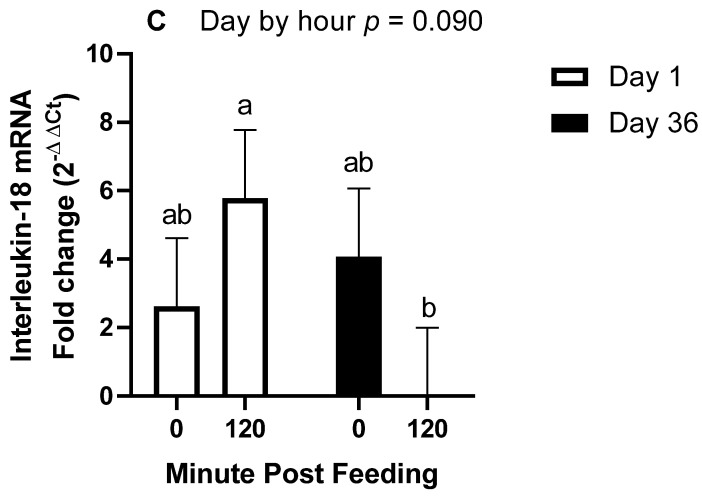
mRNA abundance of interleukin-1β (**A**), caspase-1 (**B**), and interleukin-18 (**C**) in horse white blood cells collected after an overnight fast (0 min post-feeding) and at 120 min post-feeding. Differences in transcript abundance are presented for day 1 and day 36 of barley consumption (**A**,**C**) or by age (**B**) whereby horses were either middle aged (12–14 years, *n* = 3) or older (20–22 years, *n* = 3). ^ab^ Unlike superscripts indicates geometric means differ *p* < 0.05.

The fold change abundance of IL-1β mRNA tended to be influenced by the day by hour interaction (*p* = 0.059; Figure 5), whereby transcript abundance was not different on day 1 between pre-feeding (1.2 ± 0.5) and post-feeding (1.9 ± 0.5; *p* > 0.3), while abundance on day 36 tended to be lower post-feeding (0.3 ± 0.5) than pre-feeding (1.8 ± 0.5; *p* = 0.077). Abundance was not different pre-feeding between day 1 and 36 (*p* > 0.4) but tended to be lower post-feeding on day 36 than day 1 (*p* = 0.053). The day-by-treatment and hour-by-treatment interactions were not significant (*p* > 0.4).

The fold change abundance of caspase-1 mRNA was affected by the day by hour interaction (*p* = 0.046; Figure 5), whereby transcript abundance was not different on day 1 between pre-feeding (1.1 ± 0.4) and post-feeding (1.4 ± 0.4; *p* > 0.5), while abundance on day 36 was lower post-feeding (0.2 ± 0.4) than pre-feeding (1.5 ± 0.4; *p* = 0.025). Abundance was not different pre-feeding between day 1 and 36 (*p* > 0.4) but was lower post-feeding on day 36 than day 1 (*p* = 0.044). The day-by-treatment and hour-by-treatment interactions were not significant (*p* > 0.5).

The fold change abundance of IL-18 mRNA tended to be influenced day by hour interaction (*p* = 0.093; Figure 5), whereby transcript abundance was not different on day 1 between pre-feeding (2.7 ± 1.9) and post-feeding (5.8 ± 1.9; *p* > 0.2), or on day 36 between pre (4.1 ± 1.9) and post-feeding (0.0 ± 1.9; *p* > 0.1). Abundance was not different pre-feeding between day 1 and 36 (*p* > 0.6) but tended to be lower post-feeding on day 36 than day 1 (*p* = 0.062). The day-by-treatment and hour-by-treatment interactions were not significant (*p* > 0.2).

## 4. Discussion

The main objective of this research was to determine the effects of body condition and age on the IL-1β response to ingestion of 1.2 g NSC/kg bodyweight. The main finding of this study was that when controlling for age and only using middle-aged horses, being over-conditioned was associated with a post-prandial increase in IL-1β, whereas lean horses only observed this increase after 14 days of NSC consumption. Secondly, in horses of moderate body condition, older horses had an enhanced IL-1β plasma response after 36 days of NSC consumption compared to middle-aged horses. Our third finding was the downregulation of inflammasome transcript abundance 2 h post-feeding after 36 days, which is intriguing as it contrasts the increase in plasma protein concentrations of IL-1β.

Regarding our first finding, we observed that overweight horses responded to the barley consumption differently from lean horses. We have previously reported an effect of NSC consumption to induce an immediate post-prandial elevation in IL-1β concentrations within 1 h of feeding on the first day of feeding [7,9]. In the current study’s first trial, which used horses of middle age, lean horses did not have an IL-1β response on day 1. The differences observed between the current and former studies could be due to our specifically choosing lean horses between a BCS of 4 and 5, whereas previous studies included horses with higher BCS ranges. The overweight horses responded similarly to previous reports and suggests that the day 1 post-prandial IL-1β protein response is specific to horses with higher body condition scores. Of note, the mean IL-1β concentrations increased from day 1 to day 14 in lean horses when they consumed a daily meal providing 1.2 g NSC/kg body weight per day for 14 days. These results could indicate that the capacity to produce and secrete mature IL-1β protein increases in response to regular exposure to a high-starch and -sugar meal and having a higher body condition.

The effects of overweight and obesity on inflammation are well established in human and rodent models [15]; however, equine literature has not shown consistent data. For instance, [16] did not find a difference in fasting circulating concentrations of IL-1β between equine metabolic syndrome horses (BCS 7) and healthy horses (BCS 5.75). Similarly, there was no correlation between plasma IL-1β concentration and BCS in mixed breed, sex, and aged horses that had access to hay at the time of sampling [17]. Alternatively, others have found increased adipose tissue gene expression of IL-1β in obese horses [11]. The production and release of mature IL-1β is controlled by activating inflammasomes and caspase-1, the enzyme that cleaves pro-IL-1β to its mature form [18]. Therefore, upregulation of transcript abundance will not correlate to increased plasma concentrations without activation of inflammasomes. In humans and rodent models, IL-1β is tied to the pancreatic β cell dysfunction of type II diabetes through the effect of IL-1β to inhibit glucose-stimulated insulin secretion [19]. Pancreatic β cells may also produce IL-1β in response to inflammatory stimuli. Hyperglycemia causes oxidative stress in these cells, which in turn promotes inflammasome scaffolding, caspase-1 activation, and production of IL-1β [20]. Mechanistic studies of the inflammasome in horses have received minimal attention in equine models.

An additional finding of interest came from our second trial, in which we discovered that when selecting horses of moderate body condition (BCS 5–6), older horses had an enhanced IL-1β plasma protein response to barley consumption as compared to younger horses. Higher concentrations of inflammatory proteins, even in an unstimulated state, have been documented in horses older than 20 years [10]. Similarly, LPS-stimulated IL-1β protein production is greater from elderly human white blood cells as compared to those from young humans when tested in vitro [21]. The effect of age to enhance inflammation is thought to be due to various influences, including chronic stimulation of the innate immune system throughout the lifespan and a reduced capacity of older individuals to differentiate between the severity of signals that activate inflammation [22]. Lastly, although we did not design an experiment to test the interaction of age and obesity, this may also be present and should receive further study. In the experiment by Reynolds et al. [11], which used horses averaging 17.4 to 18.8 years, adipose tissue gene expression of IL-1β was greater in obese animals compared to lean animals. Our study lends evidence to the body of knowledge regarding the effects of age on inflammatory protein production in horses.

To support our findings regarding plasma protein concentrations of IL-1β, we also investigated gene expression of *CASP1*, which is translated to caspase-1, the enzyme that cleaves pro-IL-1β to its mature form. We also investigated transcript abundances of IL-1β and IL-18. We expected that the 2 h post-feeding samples would demonstrate a higher abundance of these genes with further elevations on day 36. For instance, the protein levels of caspase-1 and IL-1β were increased in the livers of mice fed a high-fat diet [23]. In contrast, we found that all three genes exhibited reduced transcript abundance at 2 h post-feeding on day 36, with no influence of age. To our knowledge, this is the first indication of a rapid reduction in gene expression of *CASP1*, *IL-1β*, or *IL-18* following meal consumption in any species. Potential explanations involve gene degradation and inhibitors of gene transcription. Recently, it was discovered that peroxisome proliferator-activated receptor α (PPARα) induces an antisense codon *Gm15441* which then down-regulates thioredoxin interacting protein (Txnip) [24]. Expression of TXNIP protein assists with activation of caspase-1 and inflammasome scaffolding [25]; therefore, increased Txnip would reduce caspase-1. The protein, PPARα, is a nuclear receptor and transcription factor with endogenous ligands that include fatty acids. Although most commonly known for expression in the liver and adipose tissue, PPARα is also expressed in lymphocytes [26]. Brocker et al. suggested that increased fatty acid mobilization, particularly during fasting, could activate PPARα induced production of *GM15441*, which would then downregulate inflammasomes. Although we did not measure lipid metabolism in this study, previous research in horses has noted that 1.2 g NSC/kg body weight daily does induce alterations in lipid metabolism, including the reduced ability of insulin to reduce fasting plasma fatty acid concentrations [27]. Further research should include more frequent time points to confirm these data, determine the time response of gene suppression, and investigate the potential role of lipids and PPARα in inflammasome activity.

To complete the objectives of the current study, the authors used steam-rolled barley, whereas previous studies relied more heavily on molasses and steam-flaked corn. Although these two diets contributed equivalent total NSC, they did so with varying amounts of simple sugars and starch compositions. Notably, grains contain different amounts of resistant starches [28], the starch that escapes small intestinal digestion and therefore could impact the degree of starch fermentation in the hindgut. The influence of starch composition on inflammatory responses is unknown and should receive further investigation.

In conclusion, older and heavier conditioned horses appear to have an immediate inflammatory response to NSC consumption. This finding lends further evidence to the importance of limiting NSC intake in these types of horses. Alternatively, younger and leaner horses require repeated exposure for as little as 14 days to induce the same response, suggesting that age and leanness protect them against occasional spikes in NSC consumption. Further investigation should elucidate the downstream effects of IL-1β activation and whether there is a role in acute or chronic diseases. Further, we should investigate the timeline of inflammasome priming and activation in equine models.

## Figures and Tables

**Table 1:** Nutrient profiles of mixed-grass (MG) hay, ration balancer (RB), steam-rolled barley (SRB), Coastal Bermudagrass (CB) hay, and pellets fed to horses during trials 1 and 2.

Nutrient ^1^	MG Hay	RB	SRB	CB Hay	Pellet
DE, Mcal/kg	2.2	3.4	3.6	1.9	3.1
CP, %	11.8	33.8	11.7	9.7	16.3
ADF, %	38.5	8.3	7.6	39.3	16.7
NDF, %	59.6	15.1	18.9	73.6	31.2
ESC, %	5.5	7.9	3.4	1.6	4.9
WSC, %	10.6	8.3	6.2	3.9	6.2
Starch, %	1.1	6.5	60.9	2.9	14.8
NSC, %	11.7	14.8	67.1	6.8	21.0
Ca, %	0.61	4.27	0.10	0.11	1.92
P, %	0.26	1.78	0.38	0.17	1.17

^1^ Where DE = Digestible energy, CP = crude protein, ADF = acid detergent fiber, NDF = neutral detergent fiber, ESC = ethanol soluble carbohydrates, WSC = water soluble carbohydrates, NSC = non-structural carbohydrates and is the sum of starch and WSC.

**Table 2:** Accession numbers and sequences of genes analyzed.

Gene	Forward	Reverse	Accession Number	Amplicon Size
*ACTB*	GAGCAAGAGGGGCATCCTGA	GGTCATCTTCTCGCGGTTGG	100033878	184
*IL-1β*	TGATGCAGCTGTGCATTCAGT	GCACAAAGCTCATGCAGAACA	100034237	146
*CASP1*	GAGACACTGCGCCTATCCTC	GCAAGCTTAGCCAGGTCATC	100033888	122
*IL-18*	TCTAGCGGTAACCATCTCTGTG	GTCCTGGAACACTTCTCTGAAAG	100034216	149

## Data Availability

The data presented in this study are available on request from the corresponding author.

## References

[1] Schroder K., Tschopp J. (2010). The Inflammasomes. Cell.

[2] Martinon F., Burns K., Tschopp J. (2002). The inflammasome: A molecular platform triggering activation of inflammatory caspases and processing of proIL-β. Mol. Cell.

[3] Bauernfeind F.G., Horvath G., Stutz A., Alnemri E.S., MacDonald K., Speert D., Fernandes-Alnemri T., Wu J., Monks B.G., Fitzgerald K.A. (2009). Cutting edge: NF-κB activating pattern recognition and cytokine receptors license NLRP3 inflammasome activation by regulating NLRP3 expression. J. Immunol..

[4] Mariathasan S., Weiss D.S., Newton K., McBride J., O’Rourke K., Roose-Girma M., Lee W.P., Weinrauch Y., Monack D.M., Dixit V.M. (2006). Cryopyrin activates the inflammasome in response to toxins and ATP. Nature.

[5] He Y., Hara H., Núñez G. (2016). Mechanism and regulation of NLRP3 inflammasome activation. Trends Biochem. Sci..

[6] Ahn H., Kim J., Lee H., Lee E., Lee G.S. (2020). Characterization of equine inflammasomes and their regulation. Vet. Res. Comm..

[7] Suagee J.K., Splan R.K., Swyers K.L., Geor R.J., Corl B.A. (2015). Effects of high-sugar and high-starch diets on postprandial inflammatory protein concentrations in horses. J. Equine Vet. Sci..

[8] Suagee-Bedore J.K., Wagner A.L., Girard I.D. (2017). Validation of the postprandial interleukin-1β response in horses using equine-specific antibodies. J. Equine Vet. Sci..

[9] Suagee-Bedore J.K., Wagner A.L., Girard I.D. (2018). Feeding DigestaWell Buffer to horses alters the effects of starch intake on blood pH, lipopolysaccharide, and interleukin-1β. J. Equine Vet. Sci..

[10] Adams A.A., Katepalli M.P., Kohler K., Reedy S.E., Stilz J.P., Vick M.M., Fitzgerald B.P., Lawerence L.M., Horohov D.W. (2009). Effect of body condition, body weight and adiposity on inflammatory cytokine responses in old horses. Vet. Immunol. Immunopathol..

[11] Reynolds A., Keen J.A., Fordham T., Morgan R.A. (2019). Adipose tissue dysfunction in obese horses with equine metabolic syndrome. Equine Vet. J..

[12] De Fombelle A., Julliand V., Drogoul C., Jacotot E. (2001). Feeding and microbial disorders in horses: 1-effects of an abrupt incorporation of two levels of barley in a hay diet on microbial profile and activities. J. Equine Vet. Sci..

[13] Khafipour E., Krause D.O., Plaizier J.C. (2009). A grain-based subacute ruminal acidosis challenge causes translocation of lipopolysaccharide and triggers inflammation. J. Dairy Sci..

[14] Henneke D.R., Potter G.D., Kreider J.L., Yeates B.F. (1983). Relationship between condition score, physical measurements and body fat percentage in mares. Equine Vet. J..

[15] Kawai T., Autieri M.V., Scalia R. (2021). Adipose tissue inflammation and metabolic dysfunction in obesity. Am. J. Physiol. Cell Physiol..

[16] Zak A., Siwinska N., Elzinga S., Barker V.D., Stefaniak T., Schanbacher B.J., Place N.J., Niedzwiedz A., Adams A.A. (2020). Effects of equine metabolic syndrome on inflammation and acute-phase markers in horses. Domest. Anim. Endocrinol..

[17] Suagee J.K., Corl B.A., Crisman M.V., Pleasant R.S., Geor R.J., Thatcher C.D. (2011). Relationships between inflammatory cytokines, body condition, and plasma insulin in light breed horses. J. Equine Vet. Sci..

[18] Jin C., Flavell R.A. (2010). Molecular mechanism of NLRP3 inflammasome activation. J. Clin. Immunol..

[19] Ou Y., Zheng Z., Niu B., Su J., Su H. (2020). Different MAPK signal transduction pathways play different roles in the impairment of glucose-stimulated insulin secretion in response to IL-1β. Mol. Med. Rep..

[20] Ahmed A.E., Kirova D., Konantz J., Birke S., Mansfeld J., Ninov N. (2017). Distinct levels of reactive oxygen species coordinate metabolic activity with beta-cell mass plasticity. Sci. Rep..

[21] Mooradian A.D., Reed R.L., Scuderi P. (1991). Serum levels of tumor necrosis factor alpha, interleukin-1 alpha and beta in healthy elderly subjects. Age.

[22] Franceschi C., Garagnani P., Parini P., Giuliani C., Santoro A. (2018). Inflammaging: A new immune–metabolic viewpoint for age-related diseases. Nat. Rev. Endocrinol..

[23] Dixon L.J., Flask C.A., Papouchado B.G., Feldstein A.E., Nagy L.E. (2013). Caspase-1 as a central regulator of high fat diet-induced non-alcoholic steatohepatitis. PLoS ONE.

[24] Brocker C.N., Kim D., Melia T., Karri K., Velenosi T.J., Takahashi S., Aibara D., Bonzo J.A., Levi M., Waxman D.J. (2020). Long non-coding RNA Gm15441 attenuates hepatic inflammasome activation in response to PPARA agonism and fasting. Nat. Comm..

[25] Lerner A.G., Upton J.P., Praveen P.V.K., Ghosh R., Nakagawa Y., Igbaria A., Shen S., Nguyen V., Backes B.J., Heiman M. (2012). IRE1α induces thioredoxin-interacting protein to activate the NLRP3 inflammasome and promote programmed cell death under irremediable ER stress. Cell Metab..

[26] Spaner D.E., Lee E., Shi Y., Wen F., Li Y., Tung S., McCaw L., Wong K., Gary-Guoy H., Dalloul A. (2013). PPAR-alpha is a therapeutic target for chronic lymphocytic leukemia. Leukemia.

[27] Suagee J.K., Corl B.A., Swyers K.L., Smith T.L., Flinn C.D., Geor R.J. (2013). A 90-day adaptation to a high glycaemic diet alters postprandial lipid metabolism in non-obese horses without affecting peripheral insulin sensitivity. J. Anim. Physiol. Anim. Nutr..

[28] Vasanthan T., Bhatty R.S. (1998). Enhancement of resistant starch (RS3) in amylomaize, barley, field pea and lentil starches. Starch Stärke.

